# Analyzing the Essential Attributes of Nationally Issued COVID-19 Contact Tracing Apps: Open-Source Intelligence Approach and Content Analysis

**DOI:** 10.2196/27232

**Published:** 2021-03-26

**Authors:** Jan-Patrick Weiß, Moritz Esdar, Ursula Hübner

**Affiliations:** 1 Health Informatics Research Group Faculty of Business Management and Social Sciences University of Applied Sciences Osnabrueck Osnabrück Germany

**Keywords:** COVID-19, contact tracing, app, protocol, privacy, assessment, review, surveillance, monitoring, design, framework, feature, usage

## Abstract

**Background:**

Contact tracing apps are potentially useful tools for supporting national COVID-19 containment strategies. Various national apps with different technical design features have been commissioned and issued by governments worldwide.

**Objective:**

Our goal was to develop and propose an item set that was suitable for describing and monitoring nationally issued COVID-19 contact tracing apps. This item set could provide a framework for describing the key technical features of such apps and monitoring their use based on widely available information.

**Methods:**

We used an open-source intelligence approach (OSINT) to access a multitude of publicly available sources and collect data and information regarding the development and use of contact tracing apps in different countries over several months (from June 2020 to January 2021). The collected documents were then iteratively analyzed via content analysis methods. During this process, an initial set of subject areas were refined into categories for evaluation (ie, coherent topics), which were then examined for individual features. These features were paraphrased as items in the form of questions and applied to information materials from a sample of countries (ie, Brazil, China, Finland, France, Germany, Italy, Singapore, South Korea, Spain, and the United Kingdom [England and Wales]). This sample was purposefully selected; our intention was to include the apps of different countries from around the world and to propose a valid item set that can be relatively easily applied by using an OSINT approach.

**Results:**

Our OSINT approach and subsequent analysis of the collected documents resulted in the definition of the following five main categories and associated subcategories: (1) background information (open-source code, public information, and collaborators); (2) purpose and workflow (secondary data use and warning process design); (3) technical information (protocol, tracing technology, exposure notification system, and interoperability); (4) privacy protection (the entity of trust and anonymity); and (5) availability and use (release date and the number of downloads). Based on this structure, a set of items that constituted the evaluation framework were specified. The application of these items to the 10 selected countries revealed differences, especially with regard to the centralization of the entity of trust and the overall transparency of the apps’ technical makeup.

**Conclusions:**

We provide a set of criteria for monitoring and evaluating COVID-19 tracing apps that can be easily applied to publicly issued information. The application of these criteria might help governments to identify design features that promote the successful, widespread adoption of COVID-19 tracing apps among target populations and across national boundaries.

## Introduction

The COVID-19 pandemic has drawn unprecedented attention to public health measures and exposed weaknesses in governmental pandemic management efforts throughout all nations. In particular, the evidence on presymptomatic virus transmissions [1] and the large variance in severity between asymptomatic disease progression and deadly disease progression [2] has delineated the limits of centrally coordinated and executed test and trace programs. This has led to increased attention and the development of COVID-19 tracing apps for smartphones, which have been deemed as feasible and potentially effective tools for pandemic management [3]. As a result, apps are now seen as potentially important tools for supporting pandemic management; they provide a promising opportunity to complement the tracing efforts of local health authorities.

The effectiveness of contact tracing apps however depends on how many people in a given population use the app [4], which poses a unique sociotechnical challenge. A study by Trang et al [5] suggests that the large-scale willingness to use such apps is closely tied to the design factors of the app itself. This is especially true for the following design factors: privacy preservation, transparency, and convenience. Furthermore, when expanding the scope of app use to an international level, demands for transparent and open app developments become even more pressing. Contact tracing apps should not only have the ability to function in one country, but also have the ability to be interoperable with the solutions used in other countries (or the same app should be used in multiple countries), as SARS-CoV-2 spreads around the globe with little regard for national borders. At the same time, app users must be assured that a given contact tracing app is not misused as a surveillance tool by the issuing government. Such misuse might compromise human rights, privacy, and the acceptance of app use. Thus, technical app designs, especially those that relate to privacy-preserving elements, have far-reaching consequences.

The challenges of effectively using contact tracing apps are fundamentally similar across governments. This calls for the close monitoring of the different approaches to guiding governments in choosing the most promising apps. Although several studies have investigated the acceptance factors of COVID-19 tracing apps [5-8], limited research has been conducted on cross-country comparisons. O’Neill et al [9] published an overview of several national COVID-19 tracing apps. However, their study was not conducted in a scientific context, and they used a rather small set of evaluation criteria [9]. Ming et al [10] assessed various COVID-19–related apps across several countries but only briefly touched on these apps’ tracing functionalities. Furthermore, checklists and item sets that pertain to the evaluation of general mobile health apps [11-15] do not seem particularly suited for evaluating contact tracing apps, as most criteria do not apply or are too unspecific. Therefore, our goal was to develop an item set that was suitable for describing and monitoring nationally coordinated COVID-19 contact tracing apps. This item set could provide a framework for describing the key technical features of such apps and monitoring their use based on widely available information.

## Methods

In order to develop a suitable evaluation framework that accounts for feasibility in terms of the public availability of relevant information, we conducted an inductive procedure in which we screened the development and use of COVID-19 tracing apps in different countries over several months (from June 2020 to January 2021). The process of identifying and monitoring the apps was performed by using an open-source intelligence (OSINT) approach [16]. This procedure lends itself to the collection of data and information on new and emerging situations wherein there is only limited scientific knowledge available. Although this method involves drawing on scientific publications in journals, it goes beyond this and draws on a multitude of publicly available sources such as (1) grey literature (technical reports, preprints, white papers, and business documents); (2) government data (reports, press conferences, websites, and speeches); (3) conventional media (newspapers, magazines, radio, and television); and (4) the internet (web-based publications, blogs, and social media).

This approach enables the gathering of the most current information in a timely manner and helps to quickly assess the different cultural contexts of various countries. Textbox 1 provides an overview of the sources that were used in this study.

Two authors with backgrounds in medical informatics and information systems conducted parallel searches for publicly available information. Collected materials were reviewed by a third author who had a background in medical informatics. The third author also checked the collected information to determine whether the same information from different sources matched or contradicted each other. The search engines Google, DuckDuckGo, and Google Scholar were used for the search (search term combination format: “COVID-19 tracing app name” + “country name”). In addition, Wikipedia entries for COVID-19 contact tracing apps that were written in the native language of the apps’ respective countries were translated by using services that were provided by Google and Microsoft (for information that was hardcoded in pictures or fliers, the Google Translate app was used). This was done to find references for COVID-19 contact tracing apps, as Wikipedia pages that were written in the native languages of the apps’ respective countries were more up to date than those that were written in English and German (the two languages spoken by the authors). Our main information source was official information that was provided by the countries themselves (ie, technical reports from the apps’ developers or media statements that were provided by official representatives of the countries). Media and academic publications were used to verify the information that was provided by the official sources of the countries or to find information that was not provided by official sources.

**Overview of the type of sources in this study.**

**Academic publications (9 documents) [5-8,17-21]**

- Peer-reviewed information from conferences or journals
- Can also include nonpeer-reviewed sources like theses and dissertations

**Public government data (28 documents) [22-48]**

- Official sources, such as public government reports, press releases, government websites, or development repositories (eg, GitLab and GitHub)

**Grey literature (5 documents) [49-53]**

- Technical reports or preprints

**Media (11 documents) [9,54-63]**

- News and articles from newspapers, magazines, radio, television, and podcasts (eg, British Broadcasting Corporation, The Guardian, and The New York Times), especially those on the internet
- News and articles from social media websites (eg, Twitter, YouTube, etc)

The initial entry points for researching relevant information revolved around subject areas that were reported to be important design factors in previous studies [5,18-21,53] on tracing apps, such as transparency, privacy protection, data processing techniques (the protocols used and the centralization of data processing), and app features. The documents and websites that were gathered from selected countries were then analyzed by using content analysis methods, which were applied throughout several iterations to inductively extract coherent, topical categories that emerged from the available information [64]. Each category was refined into subcategories and a set of related features. These were subsequently paraphrased as question items. The final item set was then reapplied to the selected countries.

**Figure 1:**
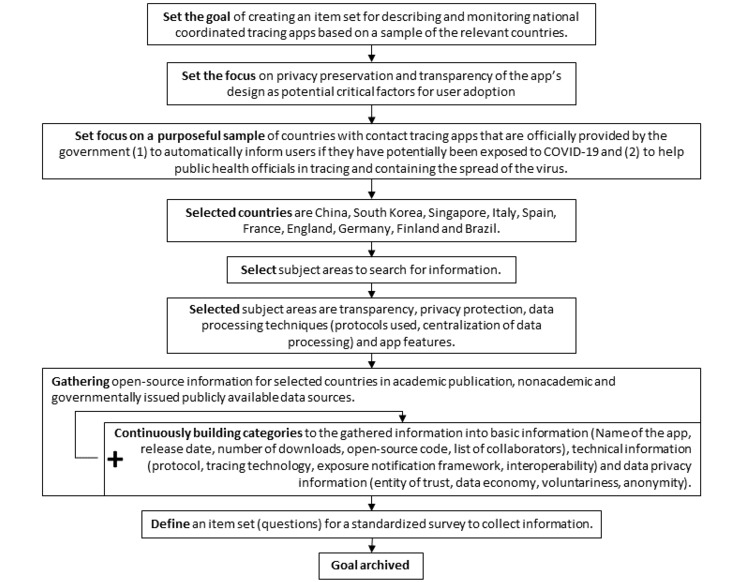
Sequence of methodological steps.

The iteration process, its findings, and the final item set framework are presented in the Results section. Figure 1 summarizes our entire methodological approach. These methodological steps were applied to information from a sample of countries with contact tracing apps that were officially provided by the government to (1) automatically inform users about whether they have potentially been exposed to COVID-19 and (2) help public health officials with tracing and containing the spread of SARS-COV-2. The sample of national tracing apps was purposefully selected; our intention was to include apps from different countries from around the world. By looking at various countries, we aimed to explore the variety and variability in the technical attributes of COVID-19 tracing apps. The nationally commissioned apps from the following countries (in alphabetical order) constituted the purposeful sample: Brazil, China, Finland, France, Germany, Italy, Singapore, South Korea, Spain, and the United Kingdom (England and Wales). These countries reflect different regions that had different experiences with the COVID-19 outbreak and the containment of the pandemic. We also expected different technological approaches across the selected countries.

## Results

### Summary of Categories

After the initial screening of the sources, several clusters of information needs emerged. These were refined and summarized into five categories (Textbox 2). These categories were then used to derive the final item set.

In the following sections, the information we collected for each category is presented. These data are based on our review of the following apps: Corona Warn App (Germany), TousAntiCovid (France), Immuni (Italy), Radar COVID (Spain), Koronavilkku (Finland), NHS (National Health Service) COVID-19 (England and Wales), TraceTogether (Singapore), Self-Diagnosis app (South Korea), Self-Quarantine app (South Korea), and Coronavirus-SUS (Sistema Único de Saúde; Brazil). Of note, China has no single app and instead uses various other apps (eg, WeChat) that have integrated tracing solutions. The order of this list mirrors the availability of information (most to least available) in the 10 selected countries.

**Categories and related information needs.**

**Background information**

- Open-source code
- How transparent is the development process?
- Is information regarding the development process and the app disseminated? If yes, how is it done?

**Purpose and workflow**

- Is the exact position of users being tracked?
- Can health authorities contact users via the app?
- Is additional data gathered (eg, for epidemiological analysis)?

**Technical information**

- Which protocol is implemented?
- Which tracing technology is used?
- Is the Google/Apple Exposure Notification System used?
- Is it interoperable with other apps?

**Privacy protection**

- Is a centralized or decentralized approach used?
- Can the gathered data be deleted?
- Does the user have to provide any personal information?
- Is the use of the app mandatory (eg, access controls)?

**Availability and use**

- When was the app released?
- How often was the app downloaded?

### Background Information

After investigating the first category (ie, background information), we found that the provision of primary background information by the issuers of the apps (Table 1) was essential for assessing the issuers’ intentions for the app and the apps’ implications for data privacy. The apps of Germany [41], France [24], Italy [38], Spain [65], Finland [66], England and Wales [67], and Singapore [23] have open-source repositories of their code bases and websites [32,34-37,42,68,69], which act as dedicated information hubs for citizens and tourists. Except for Singapore’s and South Korea’s apps, all countries’ apps were commissioned by their respective health and digital ministries. However, the apps were mainly developed, deployed, and maintained by the private industry sector. Singapore and South Korea have dedicated ministries that are capable of developing contact tracing apps with minor support from the private industry sector. Only the German development team from SAP SE (Systeme, Andwendungen, Produkte in der Datenverarbeitung, Societas Europaea) provided some form of insight into their app development process via a wide range of documents, including the scoping document, technical documentation, and an issue tracker that can be found in their publicly accessible GitHub repository [41]. There was no documentation found for the apps of Brazil, China, and South Korea (eg, documents with information about open-source code or dedicated websites). As such, various publicly available sources, like statements from press conferences, government-issued guidelines, coverage by traditional media, and social media, had to be searched to obtain information about these countries’ apps [28,48,55,57,60,70-73].

**Table 1:** Background information provided by the issuers of the apps.

App	Country	Open-source repository	Information websites
Corona Warn App	Germany	Yes	Yes
TousAntiCovid	France	Yes	Yes
Immuni	Italy	Yes	Yes
Radar COVID	Spain	Yes	Yes
Koronavilkku	Finland	Yes	Yes
NHS^a^ COVID-19	England and Wales	Yes	Yes
TraceTogether	Singapore	Yes	Yes
Self-Diagnosis app	South Korea	No	No
Self-Quarantine app	South Korea	No	No
Coronavirus-SUS^b^	Brazil	No	No
Various integrated tracing functions in apps (eg, WeChat)	China	No	No

^a^NHS: National Health Service.

^b^SUS: Sistema Único de Saúde.

### Purpose and Workflow

Our analysis of the second category (ie, the purpose of the app) showed that all of the apps that were included in this study were developed to help public health officials with tracing and containing the spread of SARS-CoV-2, as stated in the materials that were publicly accessible at the time of our search [26-28,31,32,34-37,42,48,57,60,65,68,72,74,75]. Except for the apps of China and South Korea, all apps notify their users about whether they came into contact with another user who tested positive for COVID-19. China’s app informs its users via a 3-color code system (green, yellow, or red) and uses a quick response (QR) code that can be scanned by authorities and businesses. Green indicates a person who has unrestricted movement, yellow indicates a person who must undergo a 7-day home quarantine, and red indicates a patient with confirmed COVID-19 who needs to be quarantined for 14 days. In this system, it is not clear as to how these codes are assigned and removed [55,60,71]. South Korea uses a slightly different approach compared to those of the other countries. Most apps automatically match possible COVID-19 contact events. However, South Korea allows its users to self-report their health status with the Self-Diagnosis app. If the reported symptoms are indicative of a SARS-CoV-2 infection, health authorities can intervene. If a person tests positive for COVID-19, a case officer from the local government is assigned, and the person who tested positive must report their symptoms via another app called the “Self-Quarantine app.” If people with COVID-19 leave their designated quarantine areas, they and the case officers receive an alert [57].

### Technical Information

With regard to the third category (ie, technical information), the Decentralized Privacy-Preserving Proximity Tracing (DP-3T) [49], Temporary Contact Numbers (TCN) [52], BlueTrace [22] and Pan-European Privacy-Preserving Proximity Tracing (PEPP-PT) [76] protocols were found to be used as reference implementations for the various apps (Table 2). DP-3T is used by almost all European countries except for France, which uses PEPP-PT with their own reference implementation for the Robust and Privacy-Preserving Proximity Tracing (ROBERT) protocol [51]. The protocols used for the apps of China and South Korea were not disclosed. The apps of Brazil, Finland, and England and Wales use the Google/Apple Exposure Notification System (GAEN) but do not provide any specific information about which reference protocol they used for app development. The DP-3T, TCN, BlueTrace, and ROBERT protocols all use Bluetooth-based proximity detection approaches. These Bluetooth-based proximity detection approaches use different mathematical models to calculate the likelihood of a potential exposure. The key parameters for these models are the time of being near other devices, the distance to other devices, and the results of an assessment for the contagiousness of a person with COVID-19. The sensitivity of these parameters can be adjusted by the issuer of the app, depending on the number of infections. South Korea relies on users’ self-reports, and when users are quarantined, the country relies on their GPS to alert health authorities about users leaving their quarantine zones [72]. China uses QR codes (to be able to access certain locations, these QR codes have to be scanned upon entry) and other undisclosed sensor data and metadata from smartphones [60,71,73]. Germany, Italy, Brazil, Spain, and England and Wales use the GAEN. The European Union has provided interoperability specifications for cross-border transmission chains between the approved apps that European Union states are planning to implement [29]. In October 19, 2020, the apps of Germany, Ireland, and Italy became interoperable with each other. This was made possible by the European interoperability gateway service, which will include other countries (eg, Finland) in the future [30,31]. Singapore and South Korea have dedicated ministries that are capable of developing contact tracing apps with only minor support from the private industry sector.

**Table 2:** Technical information of the apps.

App	Country	Protocol	Tracing technology	GAEN^a^	Interoperable with apps from other countries
Corona Warn App	Germany	DP-3T^b^ and TCN^c^	Bluetooth	Yes	Yes
TousAntiCovid	France	ROBERT^d^	Bluetooth	No	No
Immuni	Italy	DP-3T	Bluetooth	Yes	Yes
Radar COVID	Spain	DP-3T	Bluetooth	Yes	Yes
Koronavilkku	Finland	—^e^	Bluetooth	Yes	Yes
NHS^f^ COVID-19	England and Wales	—	Bluetooth	Yes	Yes
TraceTogether	Singapore	OpenTrace	Bluetooth	No	No
Self-Diagnosis app	South Korea	—	Reported data	No	No
Self-Quarantine app	South Korea	—	Reported data and GPS	No	No
Coronavirus-SUS^g^	Brazil	—	Bluetooth	Yes	No
Various integrated tracing functions in apps (eg, WeChat)	China	—	Quick response code, sensor data, and metadata	No	No

^a^GAEN: Google/Apple Exposure Notification System.

^b^DP-3T: Decentralized Privacy-Preserving Proximity Tracing.

^c^TCN: Temporary Contact Numbers.

^d^ROBERT: Robust and Privacy-Preserving Proximity Tracing.

^e^Not available.

^f^NHS: National Health Service.

^g^SUS: Sistema Único de Saúde.

### Privacy Protection

The different protocols have various implications with regard to the privacy of users (the fourth category). China and South Korea use fully centralized protocols and have no methods in place for anonymizing collected data (Table 3). The other apps from Germany, France, Italy, Spain, England and Wales, Singapore, and Brazil use different protocols but function similarly to each other. All countries’ apps use Bluetooth to perform tracing; they do not use any other methods of geolocation (eg, using GPS data). As such, the apps only record how long two devices (smartphones) come into contact but not where contacts occur. Singapore’s app requires mobile phone numbers to be registered. However, aside from this feature, none of the apps collect any user-identifying data and only exchange alternating IDs via Bluetooth. If the GAEN is used, the tracing and identification of contact events with people who test positive for COVID-19 are locally conducted on users’ smartphones in a decentralized manner. The apps of Singapore and France also trace contacts locally on smartphones via user logs. However, user logs must be uploaded to the centralized servers of health authorities for report processing. These servers identify contact events with people who test positive for COVID-19 and send warning messages to users. Additionally, Singapore has combined the function of its contact tracing app, TraceTogether, with the function of the SafeEntry check-in system. In this system, a QR code that contains users’ contact information must be scanned before entering a location. This information is then uploaded to a government server. England and Wales have also implemented a similar venue check-in function directly into their app.

In the decentralized approach of Germany, Italy, Spain, Finland, England and Wales, and Brazil, user logs never leave the smartphone. However, if app users from these countries test positive for COVID-19, they can enable their apps to upload a key to the central server of health authorities. This key is then sent to all devices, and health authorities can subsequently derive device IDs and check if they match one of the encounters in the user log. The use of contact tracing apps in Germany, France, Italy, Spain, Finland, England and Wales, Brazil, and Singapore is completely voluntary. Furthermore, the apps, along with their data, can be removed and deleted at any time.

**Table 3:** Privacy protection.

App	Country	Contact tracing approach	Report processing approach	User can opt out	Anonymity (no user registration)
Corona Warn App	Germany	Decentralized	Decentralized	Yes	Yes
TousAntiCovid	France	Decentralized	Centralized	Yes	Yes
Immuni	Italy	Decentralized	Decentralized	Yes	Yes
Radar COVID	Spain	Decentralized	Decentralized	Yes	—^a^
Koronavilkku	Finland	Decentralized	Decentralized	Yes	Yes
NHS^b^ COVID-19	England and Wales	Decentralized	Decentralized (tracing)/centralized (check-ins)	Yes	Yes
TraceTogether	Singapore	Decentralized	Centralized	Yes	Phone number
Self-Diagnosis app	South Korea	—	Centralized	No	Name, address, and phone number
Self-Quarantine app	South Korea	—	Centralized	No	Name, address, and phone number
Coronavirus-SUS^c^	Brazil	Decentralized	Decentralized	Yes	Yes
Various integrated tracing functions in apps (eg, WeChat)	China	Centralized	Centralized	No	No

^a^Not available.

^b^NHS: National Health Service.

^c^SUS: Sistema Único de Saúde.

### Availability and Use

With regard to the fifth category (ie, availability and use), the use of contact tracing apps first occurred in Asian countries, starting with China [70] (integrated tracing functions in existing apps like WeChat or Alipay) and South Korea [28] (Self-Diagnosis app and Self-Quarantine app) in February 2020 and Singapore [77] (TraceTogether) in March 2020. Italy [74] (Immuni), France [75] (their app originally launched as StopCovid and relaunched as TousAntiCovid in October 2020 [78]), and Germany [27] (Corona Warn App) all released their contact tracing apps in June 2020. Brazil expanded the function of its app [48] (Coronavirus-SUS) so that citizens could seek information about COVID-19 (eg, noticeable fake news and recent outbreak locations) and report possible COVID-19 symptoms to check for a potential SARS-CoV-2 infection; a tracing function was added in August 2020. Most of the autonomous communities in Spain adopted the Radar COVID app after COVID-19 testing ended in other regions at the end of August 2020 [79]. Finland also released its app at the end of August 2020 [33]. After a failed attempt at developing a contact tracing app (a result of privacy issues), England and Wales abandoned its first app development approach and released a new app in September 2020. This app also used the GAEN [25,26,54].

Although most countries in our sample regularly reported download numbers, Brazil did not provide regular reports on recent download numbers (Table 4). There are also no comparable download numbers available for China and South Korea, as China integrated their tracing functions into popular existing apps and South Korea used a different tracing system in which not all citizens continuously use one app. For South Korea however, download numbers are available from the Google Play Store; Apple does not report on the number of downloads.

**Table 4:** Availability and use.

App	Country	Release date	Downloads, n (% of population)	Date reported	Source type
Corona Warn App	Germany	June 16, 2020	25.4 million (30.6%)	February 11, 2021	Official [40]
TousAntiCovid	France	June 2, 2020	11 million (16.4%)	December 8, 2020	Official [43]
Immuni	Italy	June 15, 2020	10.3 million (17.06%)	February 22, 2021	Official [44]
Radar COVID	Spain	August 2020	7.03 million (17%)	February 14, 2021	Official [45]
Koronavilkku	Finland	August 31, 2020	>2.5 million (45.3%)	November 5, 2021	Official [46]
NHS^a^ COVID-19	England and Wales	September 24, 2020	21.8 million (36.7%)	February 10, 2021	Official [47]
TraceTogether	Singapore	March 20, 2020	4.2 million (73.7%)	February 23, 2021	Official [34]
Self-Diagnosis app	South Korea	March 2020	>500,000 (0.96%)	February 23, 2021	Google Play Store [61]
Self-Quarantine app	South Korea	March 2020	>500,000 (0.96%)	February 23, 2021	Google Play Store [62]
Coronavirus-SUS^b^	Brazil	August 1, 2020	10 million (4.77%)	December 8, 2020	Official [48] and press [63]
Various integrated tracing functions in apps (eg, WeChat)	China	February 2020	—^c^	—	—

^a^NHS: National Health Service.

^b^SUS: Sistema Único de Saúde.

^c^Not available.

### Evaluation Framework

Conducting research on the collected materials and the five categories allowed us to create more specific subcategories and derive a potential item set that was suitable for describing and monitoring nationally coordinated tracing apps (Table 5). As more information was found through the OSINT-based search, the items were phrased in a more detailed manner. This proposed item set for a monitoring framework was applied to the apps of the 10 countries that were analyzed in this study (Multimedia Appendix 1). In total, 19 items were applied to the 11 apps of the 10 countries. This yielded 209 cells of information in the matrix. However, data for 22 cells were not publicly available. These 22 cells were mainly related to information from China, but they were also somewhat related to information from South Korea, Brazil, Spain, and England and Wales. The items that were missing information were primarily related to the protocols that countries used and data privacy issues, particularly those concerning conformance with data protection regulations and the secondary use of data (other purposes).

**Table 5:** Proposed item set for a monitoring framework.

Category, subcategory, and item number				Item
**Background information**				
	**Name**			
		Q1.1	What is the name of the app?	
	**Open-source code**			
		Q1.2	Is there a publicly accessible repository of the source code?	
		Q1.3	Is the published source code up to date?	
	**Public information**			
		Q1.4	Is there some form of material (eg, website, guideline, or Frequently Asked Questions page) for informing the public about the app?	
	**Collaborators**			
		Q1.5	Which institutions worked together to develop, host, and maintain the app?	
**Purpose and workflow**				
	**Warning process design**			
		Q2.1	What is the main mode of action for warning app users?	
	**Secondary data use**			
		Q2.2	What other purposes are the data used for?	
**Technical information**				
	**Protocol**			
		Q3.1	Which tracing protocols (eg, DP-3T^a^, TCN^b^, ROBERT^c^, and BlueTrace) are implemented in the app?	
	**Tracing technology**			
		Q3.2	Which tracing technology (eg, Bluetooth, GPS, and barcodes) is used by the app?	
	**Exposure notification system**			
		Q3.3	Is the Google/Apple Exposure Notification System used?	
	**Interoperability**			
		Q3.4	Is the app actively interoperable with apps from other countries?	
**Privacy protection**				
	**Entity of trust**			
		Q4.1	Is the process of contact tracing (eg, tracking each contact event) centralized or decentralized? Provide a short description of the workflow.	
		Q4.2	Is the report processing approach (eg, matching contact events and informing the user) centralized or decentralized? Provide a short description of the workflow.	
		Q4.3	Are app data processed as mandated by data protection regulations?	
		Q4.4	Is the data automatically destroyed after a fixed period of time?	
		Q4.5	Can the user opt out?	
	**Anonymity**			
		Q4.6	Does the user have to register any information (eg, mobile phone number, name, address, or date of birth)?	
**Availability and use**				
	**Release date**			
		Q5.1	On which date could the app be downloaded by the public?	
	**Number of downloads**			
		Q5.2	Are there officially reported download numbers?	

^a^DP-3T: Decentralized Privacy-Preserving Proximity Tracing.

^b^TCN: Temporary Contact Numbers.

^c^ROBERT: Robust and Privacy-Preserving Proximity Tracing.

## Discussion

### Principal Results

COVID-19 tracing apps have become instruments in the arsenal of measures for fighting the spread of the disease. Many countries have adopted these instruments in their national pandemic management plans. This study therefore aimed to explore and identify the core features of COVID-19 tracing apps in order to provide a generic framework for conducting cross-country comparisons and monitoring apps over time. We found and screened publicly available information via an OSINT approach for analyzing selected subject areas, which served as initial entry points for collecting data. We then defined five categories to structure the evaluation framework. These categories were based on recurring information, and information for the framework was selected based on obtaining enough publicly available data. The categories were as follows: background information, purpose and workflow, technical information, privacy protection, and availability and use. Based on these categories, we constructed a set of specific items that could be used to evaluate the core features of COVID-19 tracing apps. In order to showcase the item set’s usefulness, it was applied to 10 countries that nationally commissioned COVID-19 tracing apps (ie, Brazil, China, France, Germany, Italy, Spain, Finland, Singapore, South Korea, and the United Kingdom [England and Wales]). Our comparison revealed differences among each countries’ apps, especially with regard to the centralization of the entity of trust and the overall transparency of the apps’ technical makeup. The proposed item set will help researchers evaluate the spread and use of contact tracing apps within and across countries in the future.

### Key Characteristics for Evaluating COVID-19 Tracing Apps

The application of OSINT in the emerging field of COVID-19 tracing apps heavily depends on the public availability of essential and comprehensive background information. Ideally, such information can be retrieved from an official website that informs the public and provides technical background information. Information on app development is even more useful, as open-source projects provide detailed insights and involve unrestricted code audits. In open-source projects, the source code and the entire project description are often transparently accessible. Detailed insights on app protocols and their implementation are interesting from both a technical and economic point of view (eg, increasing public acceptance); transparency likely facilitates trust, which is associated with increased public acceptance [6,7]. Our analysis revealed varying degrees of transparency across countries, as some countries disclosed most of the abovementioned information (Germany, France, Italy, Spain, the United Kingdom, and Singapore), while others provided noticeably less information (Brazil, China and South Korea).

As the motivation to download and use an app is the ultimate predictor of an app’s success, information about download numbers, acceptance, and use are the most crucial for differentiating between successful and unsuccessful app designs. According to our initial findings, relative app download numbers were highest in Singapore, Finland, the United Kingdom, and Germany. Although download numbers are indicators of motivation, they do not necessarily reveal information about an app’s actual use [17]. App use can only be measured centrally by certain methods. For instance, Germany’s COVID-19 tracing app analyzes the total number of shared negative and positive test results [50]. However, the characteristics described in our framework could be used as possible predictors of app adoption rates in cross-country adoption research.

An app’s technical design is essential not only for assessing its usefulness, but also for preserving privacy. In particular, the decision to use Bluetooth, GPS, or other means (eg, QR code scanning with corresponding protocols) determines whether the app serves the purpose of tracing or location tracking. The latter could potentially result in greater privacy concerns. Thus, tracking is unlikely to be accepted in many countries. On the other hand, several COVID-19 tracing app users have expressed their interest in obtaining more detailed information about close encounters with people with COVID-19 after receiving rather superficial information from their Bluetooth-based tracing apps [59]. This shows that COVID-19 tracing app developers face the challenge of striking the right balance between maximizing privacy preservation and maximizing usefulness (ie, the range of functionalities). It has yet to be determined which composition of app traits works best in which cultures. The use of Bluetooth is also closely related to the use of the GAEN. The GEAN system seems to be indispensable, as tracing apps that do not use it cannot run in the background of Apple/Android phones. Furthermore, many countries have shifted toward using the GAEN [58]. However, in the future, it might be desirable to find technical solutions for overcoming public health agencies’ and governments’ dependency on Google and Apple.

Another important technical feature in our item set is interoperability, which refers to an app’s ability to operate in a synchronized manner with the COVID-19 tracing apps of other countries. This has become increasingly important, given the likely resurgence of international travel when the pandemic starts to recede. Du and colleagues [80] have stated that the risk of creating a useless Tower of Babel of contact tracing apps is very real, as contact tracing apps’ inability to work across different countries renders them ineffective. Researchers should therefore identify which apps allow for cross-country use. This is particularly interesting to people who live in regions that neighbor other countries, which is quite common in Europe.

The acceptance of COVID-19 tracing apps hinges on both its perceived benefits and perceived barriers (eg, privacy concerns) [8]. The basic and technical information that are outlined in this study lay the foundation for privacy preservation. However, such information must be supplemented with data on a range of additional factors that relate to the entity of trust and anonymity. With regard to the entity of trust, we included indicators that can be used to determine whether a central or decentral data processing method is used. We found that this distinction could and should be determined for contact tracing approaches and report processing approaches. Centrality is a particularly critical factor, as the providers of COVID-19 tracing apps are often governmental agencies, and many users might be reluctant to entrust their government with the exclusive handling of health-related data. In our sample, South Korea and China stood out because they opted for approaches with higher degrees of centrality. Furthermore, the aspects of centrality and a range of other well-known criteria for privacy preservation need to be considered. Therefore, we included automatic data deletion after a fixed period of time, the possibility to opt out, and the need to refrain from storing additional personal data (eg, mobile phone numbers) into our item set.

### Limitations

Several limitations apply to our study. First and foremost, the OSINT approach depends on the public availability of reliable and trustworthy data and information. Second, the great majority of items could be answered for most, but not all, countries. This could have been due to missing information or language issues. Incorporating the insights of experts from the studied countries could have provided us with additional information and a more nuanced picture of app use/development. However, the approach we chose is more feasible, and its pragmatic nature allows for the flexible incorporation of various information sources in a quickly evolving field. Third, we developed the item set by analyzing a limited number of countries, and we cannot exclude the possibility that additional criteria would have emerged if we included additional countries in the analysis.

### Conclusion and Outlook

Ours is one of the first studies to provide a set of criteria for evaluating nationally commissioned COVID-19 tracing apps and to apply such criteria to 10 industrialized countries. Although cross-country comparisons have been previously conducted [6,9], our study provides a more comprehensive yet relatively easy-to-apply evaluation framework that uses various technical factors from publicly available sources as potential determinants of app adoption.

The evaluation of COVID-19 tracing apps is crucial for the assessment of factors that facilitate these apps’ widespread acceptance and usefulness within and across countries. The more people who accept and use the app, the better the virus can be contained. This has been demonstrated by simulation studies [4]. Given that transparency and privacy protection are crucial for building people’s trust in apps, the technical features that are proposed in our framework might play an important role in promoting widespread app adoption. The initial application of our framework to the 10 countries in this study revealed differences in countries’ app adoption rates. Based on the app download numbers, Finland, Germany, and the United Kingdom were more successful in deploying their apps because they chose transparent and decentralized contact tracing and report processing approaches. However, Singapore was the only city-state in our sample, and it had the highest app adoption rates.

The associations between technical features and app success must be addressed more thoroughly in future research. This can be done by analyzing a greater number of countries, and our framework provides the groundwork to do so. We made the item set very concise so that it can be easily shared with scientists in other countries via a collaborative approach or used to survey people in other countries via a crowdsourcing platform that is similar to that of Trang et al [5]. At the end of the pandemic, it will be interesting to see the role that apps actually played in the fight against the pandemic across different countries (ie, outside of those included in this study). Our framework will help studies that aim to evaluate apps’ contributions to the overall management of pandemics, by providing core descriptors of COVID-19 warning apps that can be used as predictors.

Insights on the success factors of tracing apps might prove useful for designing general national health apps. Over time, tracing apps might be combined with health record apps, as there have already been discussions and instances of developers expanding their apps’ features to include contact and symptom diaries, vaccination certificates, and educational resources for public health messaging.

## Appendix

Multimedia Appendix 1 Applied monitoring framework.

## Abbreviations

DP-3T: Decentralized Privacy-Preserving Proximity Tracing
GAEN: Google/Apple Exposure Notification System
NHS: National Health Service
OSINT: open-source intelligence approach
PEPP-PT: Pan-European Privacy-Preserving Proximity Tracing
QR: quick response
SAP SE: Systeme, Andwendungen, Produkte in der Datenverarbeitung, Societas Europaea
SUS: Sistema Único de Saúde
TCN: Temporary Contact Numbers
